# Hepatocellular carcinoma in children and young patients with chronic HBV infection and the usefulness of alpha‐fetoprotein assessment

**DOI:** 10.1002/cam4.917

**Published:** 2016-10-17

**Authors:** Hitoshi Tajiri, Tomoko Takano, Hideo Tanaka, Kosuke Ushijima, Ayano Inui, Yoko Miyoshi, Keiichi Ozono, Daiki Abukawa, Takeshi Endo, Stephen Brooks, Yasuhito Tanaka

**Affiliations:** ^1^ Department of PediatricsOsaka General Medical CenterOsakaJapan; ^2^ Division of Epidemiology and PreventionAichi Cancer Center Research InstituteNagoyaJapan; ^3^ Department of PediatricsKurume University Medical CenterKurumeJapan; ^4^ Department of Pediatric Hepatology and GastroenterologySaiseikai Yokohama City Tobu HospitalYokohamaJapan; ^5^ Department of PediatricsOsaka University HospitalSuitaJapan; ^6^ Department of PediatricsMiyagi Children's HospitalSendaiJapan; ^7^ Department of Pediatrics and NeonatologyNagoya City University Graduate School of Medical SciencesNagoyaJapan; ^8^ Department of Microbiology/ImmunologyState University of New York at BuffaloNew York; ^9^ Department of Virology and Liver UnitNagoya City University Graduate School of Medical SciencesNagoyaJapan

**Keywords:** alpha‐fetoprotein, HBeAg seroconversion, hepatocellular carcinoma, interferon, liver cirrhosis

## Abstract

The aims of the study were to elucidate the clinical characteristics of patients who developed hepatocellular carcinoma (HCC) related to persistent HBV infection since childhood and to investigate usefulness of assessing alpha‐fetoprotein (AFP) in this population. A nationwide multicenter survey of children with chronic HBV infection was performed. Among 548 patients, 15 patients developed HCC at the median age of 15 years (range 9–36), including 13 males and 2 females. A case–control comparison showed that HBeAg seroconversion and liver cirrhosis were associated with the occurrence of HCC. Of the 15 HCC patients, 5 were treated with interferon and none of them responded to interferon therapy as compared with 12 of the 17 responders in the control group. Of the 15 patients, 10 died and 9 of the 10 who died never visited any medical facilities until diagnosis of HCC, while the remaining 5 surviving patients never stopped their clinic visits. The usefulness of AFP assessment was shown by the findings that AFP levels were elevated in all HCC cases, that elevations in AFP levels were detected prior to the diagnosis in the surviving patients, and that sensitivity of AFP as a diagnostic test for HCC was very high among 40 patients including our 14 and an additional 26 collected from the literature. HBeAg seroconversion and liver cirrhosis are associated with the occurrence of HCC. Regular measurement of AFP might be helpful to watch for the occurrence of HCC when following children and young patients with chronic HBV infection since childhood

## Introduction

The prognosis of patients with chronic hepatitis B is unfavorable when complicated by hepatocellular carcinoma (HCC) 1, 2, 3. While there have been sporadic reports of the occurrence of HCC in childhood or early adulthood in patients with chronic HBV infection since childhood 4, 5, there has been no data on this issue derived from a nationwide multicenter survey.

The clinical features of HBV‐related HCC during childhood have been reported from Taiwan where genotype B is the most prevalent and maternal transmission was the main route of HBV infection 6, and the prognosis for most children who presented with an advanced stage was very poor 4. The reported outcome of chronic hepatitis B infection in Caucasian children has shown that liver cirrhosis was rare, but was a risk factor for HCC 7. Genotype C, which is the most prevalent genotype in Japan, has been shown to be associated with HBV‐related HCC in adults 8, 9. However, there have been no clinical studies on HCC in a young population, including children, in Japan. Only two young adults with genotype C HBV infection who presented with HCC have been reported 10. Thus, the clinical features of cases with HCC that develop during childhood, adolescence, and early adulthood, or risk factors associated with HCC in Japanese children, have not been elucidated.

The most commonly used screening tests for HCC are serum alpha‐fetoprotein (AFP) and ultrasonography. However, the results of the AFP screening in adults have not been satisfactory, with a sensitivity of 39% and specificity of 76%, when using 20 ng/mL as a cutoff value 11. The role of using an AFP measurement for screening or diagnosis of HCC has not been precisely investigated for children who developed HCC associated with chronic HBV infection.

To investigate the clinical characteristics of patients who developed HCC related to chronic HBV infection in childhood, adolescence, or young adulthood, a nationwide multicenter survey of children with chronic HBV infection was performed between 2012 and 2013. We also aimed to find baseline factors that may have an association with the occurrence of HBV‐related HCC by comparing patients with HCC to matched control patients and to evaluate the usefulness of measuring AFP for diagnosing HBV‐related HCC.

## Subjects and Methods

### Patients

The subjects of this survey were patients diagnosed with chronic HBV infection during childhood who were less than 15 years of age and who had visited 1 of the 18 participating institutes from 1989 until 2013. The demographics and clinical data of patients were collected using a questionnaire received from the 18 institutes that provide medical services to children with chronic HBV infection.

### Details of the questionnaire utilized in this study

The questionnaire included three sections of items regarding the demographics of the subjects, the natural course of chronic HBV‐related liver diseases, and the clinical outcome after medical treatment that included interferon (IFN) therapy. Responder to IFN therapy fulfilled all the following three conditions: HBeAg seroconversion, ALT normalization, and suppression of viral loads to <5.0 log copies/mL. For patients with HBeAg negative hepatitis, ALT normalization and suppression of viral loads to <4.0 log copies/mL were required. Annual laboratory data including white blood cell counts, platelet counts, hemoglobin, albumin, total bilirubin, AST, ALT, and AFP as well as HBsAg, HBsAb, HBeAg, HBeAb, and HBV DNA were requested both during the natural course of disease and during the observation period after medical intervention. The information obtained up to December 2013 was included in this study. The stage of HCC was determined according to the criteria proposed by LCSGJ 12.

### Case–control comparison study

To find baseline factors that may be associated with the occurrence of HBV‐related HCC, a case–control comparison design with five controls for each case was adopted in this study. Patients for the control group were selected by matching for two baseline factors, the year of birth (±1 year) and the duration of infection being 5 years or more. The second baseline factor was chosen based on the findings that the shortest duration of HBV infection was 5 years among the patients presenting with liver cirrhosis in this study. In total, 75 pair‐matched children were available for the 15 HCC patients to be used for the case–control comparison.

### Selection of AFP data to obtain a reference value for children with chronic HBV infection

To evaluate a role of AFP, the AFP data to be used for further analysis were selected based on three conditions: measured during the natural course of disease, obtained from patients between 3 and 18 years of age 13, and obtained when the accompanying platelet counts were greater than 150,000/*μ*L.

### Evaluation of the sensitivity of AFP in the diagnosis of HBV‐related HCC during childhood or early adulthood

Because the number of cases with HCC in our study was limited, we performed an evaluation of the literature to survey for previously published cases with HBV‐related HCC with accompanying AFP measurements at diagnosis. As a result, a total of 26 pediatric cases (historical patients) were collected from 11 reports 14, 15, 16, 17, 18, 19, 20, 21, 22, 23, 24. The combined data of the 26 historical patients and the 14 present patients were utilized to assess the sensitivity of AFP as a diagnostic test of HCC. As the AFP cutoff values for the assessment, 20 and 100 ng/mL were adopted.

### Statistical analysis

Differences in mean values and the comparison of the patients' characteristics between groups were computed using the Mann–Whitney *U* test and the Fisher's exact test, respectively. All statistical analyses were performed using the SPSS V.12.0 statistical package (Tokyo, Japan) based on two‐sided hypotheses tested with a significance level of *P* < 0.05.

### Ethical considerations

The study protocol complied with the ethical guidelines of the Declaration of Helsinki of 1975 (2004 revision) and was approved by the Ethics Committee of Osaka General Medical Center as well as by the respective organizations of each participating institute. A written informed consent was obtained from the patients or their guardians when appropriate.

## Results

### Demographic features of the study subjects

A total of 548 cases of chronic HBV infections since childhood (323 males and 225 females) were collected by this multicenter study (Table 1). The median age at the diagnosis of HBV infection was 2 years (range from birth to 15 years), and the median age at the last examination was 12 years (0–40 years). The route of infection was mother to child transmission in 70%, household contact in 12%, blood transfusion in 2%, and unknown in 16%. HBV genotype C was the most prevalent genotype in the present patients (83%). Part of the data on the distribution of the HBV genotype of the present subjects was as described previously 25.

**Table 1:** Demographic features of 548 patients with HBV infection

	*N* = 548
Sex (male/female)	323/225
Age at diagnosis of HBV infection (y)	2.5 (0.0–15.8)
Age at last visit (y)	12.7 (0.5–40.5)
Follow‐up period	7.8 (0.5–33)
Putative route of infection	
Mother to child (%)	381 (70)
Household contact (%)	66 (12)
Transfusion (%)	13 (2)
Unknown (%)	88 (16)
Underlying disease	32
Family history	
HBV‐related HCC (%)	34 (6)
HBV‐related liver cirrhosis (%)	21 (4)
Treatment (%)	127 (23)
IFN treatment	111
Lamivudine	15
Steroid	15
No treatment	421
HCC occurrence	15
Fatal outcome	10

HCC, hepatocellular carcinoma; IFN, interferon.

### Demographic features of patients who developed HCC

Fifteen patients developed HCC at the median age of 15 years (range 9–36 years) including 13 males and 2 females (Table 2). Transmission routes included mother to child transmission in 10 and nonmaternal transmission in 5 that included blood transfusion in 3 and the origin was unidentified in 2 patients. Underlying diseases were observed in 5 patients, including acute lymphoblastic leukemia in 2 and 1 each of premature birth, epilepsy, and epidermolysis bullosa hereditaria. The HBV genotype was determined in 10 of the 15 patients and all of them were infected with genotype C. At the diagnosis of HCC, median values of serum AST and ALT levels were 76 IU/mL and 50 IU/mL, respectively. Serum levels of AFP were measured in 14 of the 15 and found to be elevated in all of them. The viral load ranged from 2.2 to 6.2 log copies/mL in 5 of the 15, while it was below 2.1 in the remaining 10. An HBe antigen seroconversion was noted in all patients except one. The staging of HCC in 14 of the 15 HCC cases ranged from stages I to IV at the diagnosis of HCC. The diagnosis of liver cirrhosis was made in 7 of the 15. Five of the 15 underwent IFN therapy. Among the 15 HCC patients, 10 died due to cancer complications with a median survival time of 5 months (range 2–14 months) after the diagnosis of HCC. The 1‐year and 2‐year survival rates were 40% and 33%, respectively.

**Table 2:** Demographic features of 15 patients with HCC and comparison of the patients with fatal outcome with those who survived

	All cases(*n* = 15)	Fatal outcome*n *= (10)	Survived cases(*n* = 5)	*P* value
Age at diagnosis of HCC	15 (9–36)a	15 (11–31)a	16 (9–36)a	0.34
Sex (M:F)	13:2	8:2	5:0	1.00
Duration of HBV infection (years)	15 (9–25)a	15 (11–25)a	12 (9–24)a	0.49
Duration of observation after diagnosis of HCC (months)	7 (2–120)a	5 (2–14)a	55 (48–120)a	<0.001
Maternal transmission	10 (66%)	7	3	1.00
Underlying diseases	5 (33%)	2	3	0.25
No follow‐up in the preceding 1 year	9 (60%)	9	0	0.002
Symptomatic manifestation	9 (60%)	9	0	0.002
AST (U/L)	76 (20–454)a	151 (38–454)a	30 (20–36)a	0.008
ALT (U/L)	50 (19–376)a	60 (22–376)a	44 (27–51)a	0.09
*α*‐Fetoprotein	1031 (169–604,137)a	14,000 (191–604,137)a	311 (169–781)a	0.130
Undetectable HBV DNA	10	7	3	1.00
HBeAg seroconversion	14 (93%)	9	5	1.00
Stage of HCC (I/II/III/IV)b	3/2/0/9	0/0/0/9	4/1/0/0	<0.001
Liver cirrhosis	7 (46%)	5	2	0.57
History of IFN treatment	5 (33%)	1	4	0.170

HCC, hepatocellular carcinoma; IFN, interferon; a, median (range); b, IVA and IVB were combined into IV.

### Comparison of the patients with fatal outcome with those who survived from HCC

Nine of the 10 with fatal outcome had not visited a clinic within the year preceding the diagnosis of HCC and had presented with symptoms associated with HCC (Table 2). The remaining one was diagnosed with HCC 9 months after starting a visit to one of the participating hospitals. In contrast, the five surviving patients were regularly followed up for a median of 13 years (range, 8–24) and HCC was found as early stage disease during the follow‐up. At the diagnosis of HCC, the serum levels of AFP and AST were higher in the fatal group than in the surviving group. There were no statistical differences between the two groups regarding age, gender, and other baseline factors.

### Findings revealed by the case–control comparison study

Among the 548 cases collected by this study, 273 were confirmed to be infected with HBV for more than 5 years (one of the two matching baseline factors). There were no statistical differences between the 75 cases selected for a case–control comparison study and the remaining 198 nonmatched cases regarding gender, age, and other baseline factors (data not shown). By comparing the 15 HCC patients with the 75 control patients, it was shown that HBeAg seroconversion and liver cirrhosis was more frequently seen in the HCC group than in the control group (Table 3). Among the 15 with HCC, 5 had received IFN treatment and HCC developed afterward. There were no responders to IFN therapy in the HCC group as compared with 12 of the 17 responders in the controls. There was no significant difference between the two groups in the remaining baseline factors.

**Table 3:** Questionnaire‐based risk factor analysis for HBV‐related HCC cases (*n *= 15) and matched controls (*n* = 75)

	HCC case(%)a	Control(%)a	*P* value
Male sex	13 (86%)	48 (64%)	0.13
Age at diagnosis of HBV infection	4 (0–15)b	5 (0–14)b	0.67
Duration of HBV infection	15 (12–26)b	15 (6–25)b	0.21
Maternal transmission	10 (66%)	57 (76%)	0.51
Underlying diseases	5 (30%)	9 (12%)	0.05
Family history of HBV‐related CLD	1 (6.6%)	3 (4.0%)	0.52
Genotype of HBV			0.18
Genotype C	10 (100%)	42 (79%)	
Genotype non‐C	0	11 (21%)	
*α*‐Fetoprotein	14,000 (191–604,137)b	2.0 (1–25)b	0.13
HBeAg seroconversion	14 (93%)	48 (64%)	0.031
Presence of liver cirrhosis	7 (46%)	0	<0.001
History of IFN treatment	5 (33%)	21 (28%)	0.75
Response to IFN treatment			0.009
Responders	0	12 (71%)	
Nonresponders	5	5 (29%)	

CLD, chronic liver disease; HCC, hepatocellular carcinoma; IFN, interferon; a: numbers given are for questions that were answered, therefore they do not add up to the total numbers of cases or controls; blank responses are excluded from the analyses; b: median (range).

### Assessment of serum AFP values in the present cohort of children

**Figure 1:**
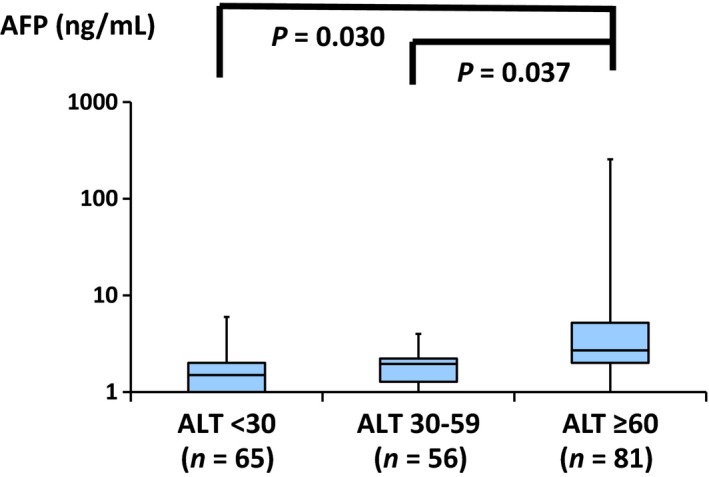
Alpha‐fetoprotein (AFP) values were sorted according to the values of ALT (IU/L): <30, 30–59, and ≥60 among the 202 non‐HCC patients. HCC, hepatocellular carcinoma. Values shown in log scale.

**Figure 2:**
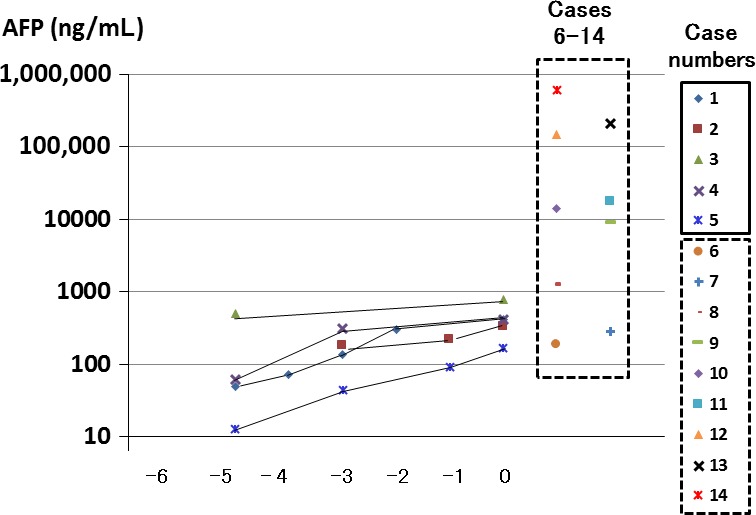
AFP values were shown serially during the preceding 5 months before the diagnosis of hepatocellular carcinoma (HCC) in the five surviving patients (cases 1–5) and at the time of diagnosing HCC in the remaining nine patients with fatal outcome (cases 6–14, confined with rectangular broken lines). AFP, alpha‐fetoprotein. Values shown in log scale.

Annual laboratory data of a set of AFP and ALT values measured using the same serum samples were obtained from 202 of the 548 patients who did not develop HCC. Both AFP and ALT were measured; a median of four times (range 1–10) and a total of 580 measurements were obtained. In this study, one AFP value, among multiple measurements, was chosen from one patient when the respective ALT value was highest. The AFP values for the 202 patients are depicted in three subgroups according to the values of ALT: <30, 30–59, and ≥60 IU/L (Fig. 1). The upper 2.5th value of AFP was 6.8 ng/mL in the subgroup with ALT <30. The median levels of AFP in the subgroup with ALT ≥60 were higher than those in the subgroups with ALT 30–59 and with ALT <30. Among the HCC patients, the serum concentrations of AFP were measured in 14 of the 15 and their AFP levels were elevated compared to those of the 202 patients in the non‐HCC group (data not shown). The AFP levels were found to be gradually increasing prior to the diagnosis of HCC in five surviving cases, whereas those in the remaining nine with a fatal outcome ranged from 191 to 604,137 ng/mL at the time of diagnosing HCC (Fig. 2). The clinical course of one of the five survivors, including serial changes in the AFP levels, was as previously reported 26.

### Assessment of the usefulness of measuring AFP for the diagnosis of HCC

Ages at diagnosis of HCC were younger in the historical patients from the literature than in the present patients (9.4 ± 3.2 vs. 18.1 ± 7.5, *P* < 0.001) and the 1‐year survival rate was substantially lower in the historical group than in the present group (1/21 vs. 6/15, *P* = 0.013). There were no differences between the two groups regarding other baseline factors (Table 4). Because the backgrounds were similar between the two groups, data of combined cases were utilized to assess the usefulness of measuring AFP in this study.

**Table 4:** Historical cases with HBV‐related HCC with accompanying AFP measurements at diagnosis

Caseno.	Age(years)	Sex	Nationality	Transmissionroutes	HBeAgSC	AFP(ng/mL)	Livercirrhosis	Pulmonary metastasis	Outcome	Last visitafter Dx (months)	Ref.
1	10	M	Thai	MT	ND	118,000	―	ND	Alive	3	14
2	4	M	Japan	MT	+	2100	―	ND	Dead	4	15
3	6	M	Japan	BT	+	2,240,000	―	ND	Dead	1	16
4	7	M	Taiwan	MT	ND	Elevated	ND	ND	Dead	8	17
5	14	M	Japan	MT	ND	4600	―	ND	Dead	2	18
6	11	M	Japan	ND	+	1225	+	ND	Dead	1	18
7	6	M	Japan	MT	ND	7,000,000	+	Present	Dead	6	18
8	10	M	Japan	MT	ND	7500	―	Present	Dead	3	18
9	9	M	Taiwan	MT	ND	Elevated	ND	ND	Dead	1	19
10	7	M	Taiwan	MT	ND	Elevated	―	ND	Dead	5	19
11	5	M	Taiwan	MT	ND	6400	+	Present	Dead	15	19
12	7	M	Taiwan	MT	ND	3200	+	Present	Dead	3	19
13	7	M	Belgium	MT	+	1,400,000	+	ND	Dead	1	20
14	6	M	Japan	MT	+	1,200,000	+	ND	Dead	ND	21
15	10	M	Japan	MT	+	51,000	+	ND	Dead	ND	21
16	12	F	Japan	MT	+	430,000	+	ND	Dead	ND	21
17	13	M	Japan	ND	ND	100,000	+	ND	Dead	ND	21
18	11	M	Italy	BT	+	800,000	ND	Present	Dead	5	22
19	7	F	German	MT	+	WNL	ND	ND	Dead	2	23
20	12	M	German	ND	+	200,000	ND	Present	Dead	2	23
21	14	M	German	MT	+	WNL	ND	ND	Dead	3	23
22	14	M	Turkey	ND	+	233,000	ND	Present	Dead	6	23
23	16	M	German	BT	+	121,000	ND	Present	Dead	1	23
24	11	M	Italy	MT	+	Elevated	+	ND	Dead	1	24
25	11	M	Italy	MT	+	800,000	―	Present	Dead	10	24
26	6	M	Italy	MT	+	1,245,000	+	ND	Dead	1	24

SC, seroconversion; Dx, diagnosis; MT, maternal transmission; BT, blood transfusion; ND, not described; HCC, hepatocellular carcinoma; AFP, alpha‐fetoprotein; WNL, normal value for AFP, <20 ng/mL.

To evaluate the performance of AFP in the diagnosis of HBV‐related HCC among children and young patients, we have set the upper limit at 20 ng/mL as recommended previously 11. The sensitivity of AFP as a diagnostic test for HCC was similar between the 14 of the present 15 patients and the 26 historical patients (data not shown). Therefore, the combined data of the 40 patients were utilized to assess the usefulness of AFP. The sensitivity of AFP was 95% and positive predictive value was 86% (Table 5A). When the upper limit of 100 ng/mL was adopted, the sensitivity was 94% and positive predictive value was 97% (Table 5B).

**Table 5:** Diagnostic accuracy of AFP for detecting HCC in the patients with childhood‐onset chronic HBV infection

*α*‐fetoprotein	HCC		
	Present	Absent	Total
(A) AFP cutoff value 20 ng/mL			
<20 ng/mL	2	196	198
≥20 ng/mL	38	6	44
Total	40	202	242
(B) AFP cutoff value 100 ng/mL			
<100 ng/mL	2	201	203
≥100 ng/mL	34	1	35
Total	36	202	238

Sensitivity (38/40), 97.0%; positive predictive value (38/44)

Sensitivity (34/36), 94%, positive predictive value (34/35), 97%

AFP, alpha‐fetoprotein; HCC, hepatocellular carcinoma.

## Discussion

Among the 548 patients included in this study with chronic HBV infection, HBV‐related HCC was observed in 15 patients during childhood, adolescence, or young adulthood. The remarkable findings of the 15 HCC patients shown by this study include a high rate of the presence of liver cirrhosis, a poor prognosis, a promising role of follow‐up in improving the prognosis of HCC, and the usefulness of using AFP levels in the diagnosis of HCC during the follow‐up. In particular, the impact of regularly measuring AFP was highlighted by the present findings that serum levels of AFP were elevated in all HCC cases, that AFP levels measured at the diagnosis of HCC were higher in the fatal group than in the surviving group, and that AFP levels had gradually increased prior to the diagnosis of HCC in the five surviving cases. This study also showed that the sensitivity of AFP as a diagnostic test for HCC was very high among the 40 patients, including both the 14 from our cohort and the 26 historical patients.

The case–control analysis of the present study showed that liver cirrhosis was significantly associated with the occurrence of HCC. Liver cirrhosis was observed in 46% of the 15 HCC cases in this study. One retrospective study has shown a high prevalence of cirrhosis in children (74%) who presented with HBV‐associated HCC 27. A recent study has also reported that 46% of Taiwanese children had liver cirrhosis as a complication at diagnosis of HCC 28. Those findings were consistent with this study and suggest that liver cirrhosis is a risk factor for HCC in children and young patients as well as in adults.

Our case–control study also showed a higher rate of HBeAg seroconversion (SC) in patients with HCC than in the matched controls. Mutations in the core/core promoter genes have been reported to be associated with HCC and such mutations were found more frequently in patients with HBeAg SC than in HBeAg‐positive carriers 29, 30. Other genetic alterations in the genomes of HBV, including mutations in cancer‐associated genes and integration of HBV DNA into host cellular DNA sequences, were found in the cells retrieved from the tumors of HCC patients 31.

A favorable response to IFN therapy with sustained effects for achieving ALT normalization and HBeAg seroconversion and for reducing the viral load in children with chronic hepatitis B has been recently shown in one study with a median observation period of 8 years 32. Importantly, the case–control analysis of the present study has also suggested a potential effect of IFN therapy on reducing the occurrence of HCC; a finding which may be worth further studies because it is critically important to determine whether IFN therapy should be recommended to reduce the future risk of HCC for children with chronic hepatitis B. Such a beneficial effect has been shown for adults 33.

A very poor prognosis for children with HCC has been reported by two single‐center studies: 2‐year survival rates being 4.1% in Taiwan 4 and 4.4% in China 28. The 2‐year survival rate in our study was 33.3%, higher than in those two studies. In our study, the factor most closely related to the prognosis of HCC was the early detection of HCC by regular follow‐up at the out‐patient clinic. Nine of the 10 patients who died of HCC were not followed by any medical facilities and were diagnosed when symptoms of HCC manifested. In contrast, HCC was diagnosed at the early and asymptomatic stage during regular follow‐up in the five survivors. Early detection of HCC by constant out‐patient clinic visits could lead to successful surgical resection of cancer that represents the best hope of long‐term survival 4.

Universal newborn vaccination has decreased the occurrence of HCC from childhood to early adulthood 34, 35. Failure to prevent HCC results mostly from unsuccessful control of HBV infection from highly infectious mothers 34. Nowadays, universal newborn vaccination to prevent HBV infection during childhood is implanted in 93% of countries and areas in the world. A newly launched global campaign against HBV‐related HCC in young people can be implemented with early detection of HCC by a nationwide surveillance system with AFP and ultrasonography. In fact, in Japan, a visit to medical facilities is discontinued in many patients with chronic HBV infection during adolescence or in young adulthood because the patients' knowledge about the morbidities and complications associated with chronic HBV infection is not sufficient. To provide continuous care to young patients who contract HBV infection during childhood and carry a lifetime risk of HCC, it is urgently needed to institute a system to insure continuous care during the bridging period from pediatricians to adult hepatologists and also to develop systems that promotes the education of young patients and their families as well as primary medical care providers.

In this study, the usefulness of AFP was assessed among children with chronic HBV infection. The upper 2.5th value of AFP was figured to be 6.8 ng/mL in the subset of the patients with normal ALT levels. In the previous study, the reference values for AFP for healthy children between 3 and 18 years of age ranged from 0.6 to 2.0 ng/mL 13. The difference between the two reference values may be due to the background of each study population; this study was planned to collect the annual laboratory data and may have failed to cover periods with hepatitis accompanied by elevated levels of AFP. This variable never occurred in the latter study that excluded the patients who were sick.

There have been no studies that evaluated the usefulness of following AFP levels for diagnosis of the development of HCC in HBV‐infected children. However, in adults the usefulness of AFP as a screening test has been evaluated 11, 36. The review on this issue summarized that the sensitivity ranged from 39% to 64% and the specificity ranged from 76% to 91% when an upper limit of AFP of 20 ng/mL was used. If the level of AFP was increased from 20 to 100 ng/mL, the sensitivity of AFP decreased from 36% to 13%, while the specificity increased from 76% to 97% 11. In this study, the sensitivity of AFP as a diagnostic test for HCC was 95%, whether using 20 or 100 ng/mL as a cutoff value. A better performance of AFP in the present cohort that included children, adolescences, and young adults than in the previous studies of adults might be due to the difference in the duration of HBV infection among the studies. As screening tests for HCC, various examinations including AFP and ultrasonography are recommended. Among those, serum AFP may have an advantage by being one of the routine laboratory tests having a relatively low cost. Our results suggest that the usefulness of AFP as a screening test in a young population with chronic HBV infection may be worth future prospective studies.

Several limitations exist in this study. First, all of the patients with fatal outcome were found at Stage 4. This factor obviously affects our analysis of the clinical differences between the patients with fatal outcome and those who survived from HCC described in Table 2. The results obtained from a comparison between two groups of HCC provides a weak support for conclusions that in the former group symptomatic manifestation was more frequently seen and the serum levels of AST at the diagnosis of HCC were more elevated. Second, because the number of cases with HCC in our study was limited, we combined data of the 14 present patients and the 26 historical patients to evaluate the usefulness of AFP in the diagnosis of HBV‐related HCC during childhood or early adulthood. The historical group includes only patients with definite diagnosis of HCC, thus allow us to analyze only the sensitivity of AFP as a diagnostic test of HCC.

In conclusion, our data suggest that HBeAg seroconversion and liver cirrhosis are associated with the occurrence of HCC and that a surveillance system with regular measurement of AFP to watch for the occurrence of HCC may be mandatory for children and young patients with chronic HBV infection.

## Conflict of Interest

None declared.
